# Molecular Mechanism Underlying Mechanical Wounding-Induced Flavonoid Accumulation in *Dalbergia odorifera* T. Chen, an Endangered Tree That Produces Chinese Rosewood

**DOI:** 10.3390/genes11050478

**Published:** 2020-04-28

**Authors:** Ying Sun, Mei Gao, Seogchan Kang, Chengmin Yang, Hui Meng, Yun Yang, Xiangsheng Zhao, Zhihui Gao, Yanhong Xu, Yue Jin, Xiaohong Zhao, Zheng Zhang, Jianping Han

**Affiliations:** 1 Institute of Medicinal Plant Development, Chinese Academy of Medical Sciences and Chinese Peking Union Medical College, Beijing 100193, China; sunying043@163.com (Y.S.); gaomei1121@163.com (M.G.); cmyang@implad.ac.cn (C.Y.); zhgao@implad.ac.cn (Z.G.); yhxu@implad.ac.cn (Y.X.); yjin@implad.ac.cn (Y.J.); xhzhao@implad.ac.cn (X.Z.); 2 Department of Plant Pathology and Environmental Microbiology, The Pennsylvania State University, University Park, PA 16802, USA; sxk55@psu.edu; 3 Hainan Branch Institute of Medicinal Plant Development, Chinese Academy of Medical Sciences and Peking Union Medical College, Haikou 570311, China; huiziqq@163.com (H.M.); yangyun43@aliyun.com (Y.Y.); xiangsheng437@163.com (X.Z.)

**Keywords:** *Dalbergia odorifera*, flavonoid biosynthesis, heartwood, mechanical wounding, signals, transcriptome

## Abstract

*Dalbergia odorifera*, a critically endangered tree species, produces heartwood containing a vast variety of flavonoids. This heartwood, also known as Chinese rosewood, has high economic and medicinal value, but its formation takes several decades. In this study, we showed that discolored wood induced by pruning displays similar color, structure, and flavonoids content to those of natural heartwood, suggesting that wounding is an efficient method for inducing flavonoid production in *D. odorifera*. Transcriptome analysis was performed to investigate the mechanism underlying wounding-induced flavonoids production in *D. odorifera* heartwood. Wounding upregulated the expression of 90 unigenes, which covered 19 gene families of the phenylpropanoid and flavonoid pathways, including PAL, C4H, 4CL, CHS, CHI, 6DCS, F3’5’H, F3H, FMO, GT, PMAT, CHOMT, IFS, HI4’OMT, HID, IOMT, I2’H, IFR, and I3’H. Furthermore, 47 upregulated unigenes were mapped to the biosynthesis pathways for five signal molecules (ET, JA, ABA, ROS, and SA). Exogenous application of these signal molecules resulted in the accumulation of flavonoids in cell suspensions of *D. odorifera*, supporting their role in wounding-induced flavonoid production. Insights from this study will help develop new methods for rapidly inducing the formation of heartwood with enhanced medicinal value.

## 1. Introduction

The most precious rosewood is Chinese rosewood, which corresponds to the heartwood of *Dalbergia odorifera* T. Chen. This heartwood has been used for manufacturing luxury furniture, artifacts, and musical instruments due to its peculiar scent, distinctive color, and high density [1,2]. Sesquiterpenes and flavonoids mainly cause its scent and color, and its flavonoids also exhibit diverse biological and pharmacological activities, including anti-inflammatory, antioxidant, antiplatelet aggregation, angiogenic, antitumor, antimicrobial, and vasodilatory effects [3,4,5,6,7,8,9,10].

In China, the rosewood of *D. odorifera*, a medium-sized evergreen tree indigenous to Hainan Province in southern China [11], is valued as a traditional Chinese medicine for the treatment of cardiovascular diseases, blood disorders, and ischemia, as well as pain relief [12]. However, wild sources of Chinese rosewood are seriously threatened, owing to the slow and infrequent formation of rosewood and overexploitation. As a result, *D. odorifera* was put on the International Union for Conservation of Nature (IUCN) red list of endangered species in 1998 and the Appendix II list of the Convention on International Trade in Endangered Species (CITES) of Wild Fauna and Flora in 2017 [13,14]. Recently, *D. odorifera* was introduced to subtropical areas in Guangdong, Guangxi, and Fujian Provinces, with the plantation area of *D. odorifera* exceeding 3500 ha^2^. However, because *D. odorifera* is slow growing and takes approximately 50 years to form heartwood under natural conditions [15], cultivation of *D. odorifera* trees still cannot meet the demand for heartwood. New methods for rapidly producing heartwood with great medicinal value are urgently needed. 

Plant secondary metabolites have been used for producing functional foods, dietary supplements, pharmaceuticals, cosmetics and agrochemicals [16]. Plants subjected to abiotic stresses, including wounding, synthesize secondary metabolites [17,18,19] presumably as part of defense mechanisms. Accordingly, wound-induced production of secondary metabolites can potentially be exploited as a strategy for producing high levels of bioactive compounds [18,20,21]. We reported that mechanical wounding of *D. odorifera* induced the production of discolored wood that displays a color similar to that of heartwood [22]. This result suggested the possibility of exploiting wounding-induced production of bioactive compounds in *D. odorifera*.

Here, we investigated the mechanism of wound-induced heartwood formation by analyzing and comparing transcriptomes in healthy and wounded samples via high-throughput RNA sequencing (RNA-Seq). This functional genomics tool enables comprehensive and cost-effective analyses of gene expression patterns in many non-model plant species [23]. We annotated the putative functions of differentially expressed genes in wound-induced heartwood using multiple publicly available databases. In addition, changes in signaling molecules, such as hormones, and the expression of phenylpropanoid and flavonoid pathway genes in response to wounding were studied. The resulting data helped understand the regulation of flavonoid biosynthesis in *D. odorifera*. This understanding is crucial for guiding the development of novel strategies for producing specific flavonoid compounds, such as metabolic engineering and application of specific signal molecules to regulate the production of desired flavonoids. Metabolic engineering of crops such as potato [24], rice [25], and tomato [26] to produce specific compounds have been demonstrated.

## 2. Materials and Methods

### 2.1. Plant Materials

Five-year-old *D. odorifera* saplings were grown in a greenhouse at the Institute of Medicinal Plant Development in Beijing, China under day/night temperatures of 28 ± 2 °C/22 ± 3 °C. The saplings were approximately 1.5 m in height with the stem diameter of 1.5 ± 0.1 cm at 50 cm above the ground. Twenty-four-year-old *D. odorifera* trees with natural heartwood were planted in Xinyi County, Maoming City, Guangdong Province, China. Cell suspension cultures of *D. odorifera* were established via the following steps. After disinfecting fresh young leaves using a sodium hypochlorite solution, they were washed with sterile water. The disinfected leaves were cut into pieces and placed in tubes containing Murashige-Skoog, amended with Phytagel (Sigma), 2.0 mg/L, 2,4-dichlorophenoxyacetic acid, and 0.2 mg/L kinetin. After 12 subcultures, energetic and loose calli, which were appropriate for cell suspension culture, were shaken in liquid MS medium containing 0.2 mg/L 2,4-D, 0.2 mg/L 6-benzylaminopurine, and 0.5 g/L casein hydrolysate to establish cell suspension cultures.

### 2.2. Chemicals and Reagents

Fisetin, formononetin, daidzein, liquiritigenin, salicylic acid, and ethephon (purity >98%), were purchased from Sigma-Aldrich Company Ltd. (UK). Luteolin was purchased from the National Drug Reference Standards (Beijing, China). D4-SA (purity >99%), which was used as an internal standard, was acquired from Sigma-Aldrich Company Ltd. (UK). Naringenin, Jasmonic acid, and abscisic acid (purity >98%) was purchased from Aladdin Industrial Corporation. (USA). Acetonitrile (HPLC grade) and methanol (HPLC grade) were purchased from Fisher (USA). Other reagents and solvents (analytical grade) were purchased from Beijing Chemical Works (Beijing, China). Purified water using a Millipore Milli Q-Plus system (Millipore, Bedford, MA, USA), was used for making solutions.

### 2.3. Histological Observation of D. odorifera Stems

**Figure 1:**
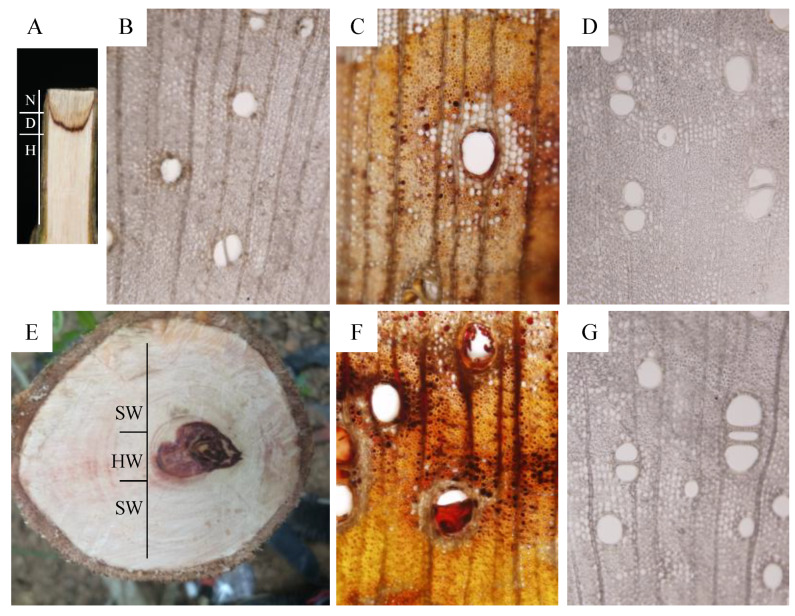
Histological observation of pruning-induced and naturally-produced heartwood samples of *Dalbergia odorifera*. (**A**) tangential section of a five-year-old *D. odorifera* sapling showing pruning-induced discoloration; (**B**–**D**) light microscope images of the transverse sections of the necrotic (N), discolored (**D**), and healthy (H) zones, respectively; (**E**) transverse section of a stem of 24-year-old *D. odorifera* tree showing natural heartwood (HW); (**F**) transverse section of natural heartwood; (**G**) transverse section of sapwood (SW).

Pruning was done by cutting stems at approximately 50 cm above the ground. After three weeks of pruning, the pruned stem was divided into three zones labeled as N, D, and H zones (Figure 1A). Subsequently, 60-µm thick sections were sliced from the N, D, and H zones using a freezing microtome (Leica CM1900, Germany). Transverse sections were cut from each sample, mounted in water on a coverslip and examined using light microscopy (BX51, Olympus, Tokyo, Japan). For each section, five areas were randomly selected for observation, as previously described [5].

### 2.4. Measurement of Total Flavonoids Content 

Total flavonoids were quantified as previously described [27,28]. Each sample (0.2 g) (filtered through a sieve with a bore diameter of 0.425 mm) was placed in a 100-mL conical flask with a glass stopper. After adding 20 mL 70% ethanol, the solution was subjected to ultrasonic vibration (pulse energy 60 kHz) for 60 min at 25 °C, shaken thoroughly, filtered and made up to 25-mL with 70% ethanol. Then, 1 mL of filtered solution and 1 mL of the NaOMe were placed into 5-mL volumetric flasks and made up with 70% ethanol solution. After 40 min at room temperature, absorbance at 410 nm was measured. The standard curve was built using the following formula: Y = 0.0209 X − 0.0005, R^2^ = 0.998, with X being the absorbance of sample and Y being the concentration of the naringenin. The linear range was 0.004–0.028 mg/mL. 

### 2.5. Simultaneous Determination of Six Flavonoids in D. odorifera Using HPLC

Six major flavonoids in *D. odorifera* were extracted and quantified as previously reported [29] with slight modifications. Powdered samples as described above (filtered through a sieve with a bore diameter of 0.425 mm; 0.1 g) in a 10-mL volumetric flask were extracted with 60% methanol in an ultrasonic bath (pulse energy 60 kHz) for 60 min. The total volume of the extract was adjusted to 10 mL with 60% methanol. This solution was filtered through a membrane filter (0.22 μm pore size) prior to injection. A Shimadzu Prominence-i LC-2030c HPLC system, equipped with a quaternary solvent delivery system, an autosampler, and UV detector (Shimadzu, Japan), was used. A Zorbax SB-C_18_ column (250 mm × 4.6 mm, 5 µm) set at 40 °C was employed for all analyses. Detection wavelength was set at 275 nm. The mobile phase consisted of (A) acetonitrile and (B) 0.3% aqueous acetic acid (v/v) using a gradient elution of 26% A at 0–16 min and 26–40% A at 16–65 min. Re-equilibration between individual runs was 15 min. The flow rate was 0.8 mL/min, and 10 μL was injected. The standard curve for each compound was generated as follows: fisetin (Y = 17,040 X + 2224, R^2^ = 0.9998, with a linear range of 0.875–35 μg/mL); daidzein (Y = 75,875 X + 1951, R^2^ = 0.9998, with a linear range of 1.209–48.375 μg/mL); liquiritigenin (Y = 39,236 X + 3018.8, R^2^ = 0.9998, with a linear range of 4.312–172.5 μg/mL); luteolin (Y = 62,191 X + 4598.9, R^2^ = 0.9998, with a linear range of 0.962–38.5 μg/mL); naringenin (Y = 49,914 X + 2333.4, R^2^ = 0.9998, with a linear range of 3.8–152 μg/mL); and formononetin (Y = 91,754 X − 7880.3, R^2^ = 0.9998, with a linear range of 0.787–31.5 μg/mL) (X refers to the concentration of the flavonoid (μg/mL), Y refers to the peak area).

### 2.6. Quantification of Plant Hormones and H_2_O_2_ in the D and H Zones

#### 2.6.1. SA, ABA, and JA 

These phytohormones in crude plant extracts were quantified using UPLC-MS/MS as previously described in [30]. Each sample was ground to fine powder in the presence of liquid nitrogen and transferred to a 2 mL Eppendorf tube containing 100 μL 1 μg mL^−1^ internal standards (2-hydroxybenzoic acid-[^2^H_4_], d_4_-SA) and 500 μL extraction solvent (2-propanol/H_2_O/concentrated HCl = 2:1:0.002, vol/vol/vol). The sample tubes were placed in a shaker at 100 rpm at 4 °C for 30 min. After adding 1 mL dichloromethane, they were shaken for another 30 min. The mixture was centrifuged at 13,000 rpm and 4 °C for 5 min, and approximately 900 μL of the lower phase was transferred to a new tube. After concentrating each collected material using an N-Evap system, it was dissolved in 200 μL methanol. Chromatographic analyses were performed using an ACQUITY UPLC H Class system coupled to a triple-quadrupole Xevo-TQD mass spectrometer equipped with an electrospray ionization Z spray™ source (Waters, Milford, MA, USA). Samples (2 μL) were injected into an ACQUITY UPLC® BEH C18 reversed-phase column (2.1 × 100 mm, 1.7 μm) purchased from Waters (Milford, MA, USA). 

#### 2.6.2. H_2_O_2_

The H_2_O_2_ concentration was measured as previously described [31,32]. Then, 30 mg of finely ground sample was mixed with 100 μL assay reagent (500 μM ammonium ferrous sulfate, 50 mM H_2_SO_4_, 200 mM xylenol orange, and 200 mM sorbitol). The absorbance at 560 nm was measured after 30 min incubation at room temperature. The specificity for H_2_O_2_ detection was tested by eliminating H_2_O_2_ in the mixture using catalase. Standard curves of individual samples were generated by adding variable amounts of H_2_O_2_ to 100 μL double distilled water and to 100 μL assay reagent. Data were normalized and expressed as mM H_2_O_2_ per gram of stem (fresh weight). 

#### 2.6.3. Ethylene

Each sample was placed in sealed 2-mL bottle capped with a rubber stopper for 1 h. Ethylene was measured by withdrawing 0.5 mL headspace air from the bottle. The air sample was injected into an Agilent 7890A gas chromatograph (Agilent Technologies, Palo Alto, California, USA) fitted with an HP-5MS capillary column (30 m × 0.25 mm id, film thickness 0.25 μm). The injector, detector, and oven temperatures were 110, 140, and 90 °C, respectively.

### 2.7. Transcriptome Analysis of Zones D and H

All clean reads generated by Illumina sequencing have been deposited in the Sequence Read Archive (SRA) data base (http://www.ncbi.nlm.nih.gov/sra) under the accession ID SRX7899170 for H, and SRX7899171 for D, and the accession number is PRJNA612155. Total RNAs from zones D and H were extracted using the Easyspin RNA reagent (RN38; Aidlab Biotechnologies, Beijing, China) according to the manufacturer’s protocol. RNA samples (RNA integrity number (RIN) ≥ 8) were used to construct cDNA libraries. Sequencing using Illumina HiSeq 4000 was performed by Novogene Bioinformatics Technology Co., Ltd. (Beijing, China). For functional annotations, all assembled unigenes were used for homology searches against the NCBI nonredundant (NR) database using an E-value cut-off of 10^−5^. After sequence assembly, the unigenes were aligned to protein databases, such as Swiss-Prot, the Kyoto Encyclopedia of Genes and Genomes (KEGG), the Clusters of Orthologus Groups (COG), and the Gene Ontology (GO) database, which were searched via BLASTX using the unigenes and the best alignment results were used for identification. Differentially expressed of unigenes between the D and H zones were identified using edgeR software as previously described [33,34,35]. A heat map was generated to note significantly altered gene expression patterns between the D and H zones.

### 2.8. Plant Hormone Treatment and Flavonoid Analysis

Methyl salicylate, methyl jasmonate, ethephon, and (±)-cis, trans-abscisic acid were added at a final concentration of 1.0 mM to seven-day-old suspension cell culture of *D. odorifera*. After incubating the treated cell suspension cultures in the dark at 25 °C for two days, they were centrifuged at 1000 rmp for 1 min. The precipitate was frozen immediately using liquid nitrogen. Each sample was ground to a fine powder in the presence of liquid nitrogen and transferred to a 2 mL Eppendorf tube containing 500 μL extraction solvent (2-propanol/H_2_O/concentrated HCl = 2:1:0.002, vol/vol/vol). The tubes were placed in a shaker at 100 rpm at 4 °C for 30 min. After adding 1 mL dichloromethane in each tube, they were shaken for another 30 min. After centrifugation at 13,000 rpm and 4 °C for 5 min, approximately 900 μL of the lower phase was transferred to a new tube. After drying each sample in an N-Evap system, it was dissolved in 200 μL methanol. Six major flavonoids were quantified using HPLC as described above.

### 2.9. Statistical Analysis

Data was calculated based on combined averages from three individual samples (n = 3). The significance of differences among data sets was determined using Duncan’s multiple comparison test at the 0.05 significance level.

## 3. Results

### 3.1. The Discolored Wood of D. odorifera Induced by Pruning Displays Structural Similarity to That of Natural Heartwood

Stem discoloration was observed three weeks after pruning of five-year-old *D. odorifera*. The pruned stem was divided into three zones, labeled as necrotic (N), discolored (D), and healthy (H), from the cut surface (Figure 1A). Structural features of these zones were analyzed. In the N zone, ray parenchyma cells and axial necrosis and fragmentation were observed, and some fungal hyphae were present in the vessel lumens of the secondary xylem (Figure 1B). In zone D, ray and axial parenchyma cells, as well as the vessels and wood fibers, were filled with brow or dark-brown resin (Figure 1C). Because these features are similar to those observed in natural heartwood (Figure 1E,F), the D zone appears to contain heartwood substances. The H zone showed a structure similar to that of yellowish-white sapwood of *D. odorifera*, where vascular occlusions were absent in the vessels (Figure 1D,G).

### 3.2. The Flavonoids Profiles in Zone D are Similar to Those in Natural Heartwood

**Figure 2:**
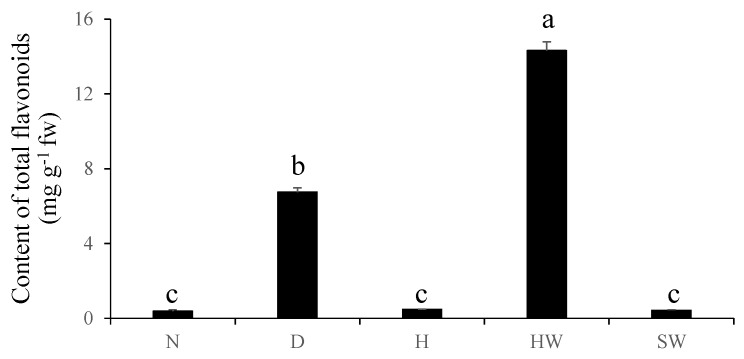
The total contents of total flavonoids in the N, D, and H zones of pruned stem, natural heartwood (HW), and sapwood (SW).

Extracts from the N, D, and H zones of pruned stem, natural heartwood (HW), and sapwood (SW) were analyzed to determine the content of total flavonoids (CTF). The CTF closely correlated with the color and luster of analyzed samples (Figure 1 and Figure 2). The HW of a 24-year-old tree had the highest CTF (14.31 mg/g), and the next highest CTF was found in the D zone (6.75 mg/g). In contrast, almost no flavonoids were detected in the N and H zones and SW (Figure 2).

**Figure 3:**
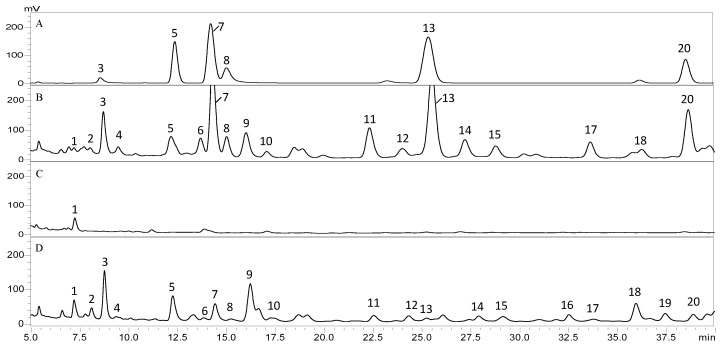
HPLC chromatograms of flavonoid in different samples. The standard mixture of (3) fisetin, (5) daidzein, (7) liquiritigenin, (8) luteolin, (13) naringenin, and (20) formononetin is shown in (**A**). These flavonoids present in (**B**) natural heartwood and the (**C**) H and (**D**) D zones of a pruned stem are shown.

**Figure 4:**
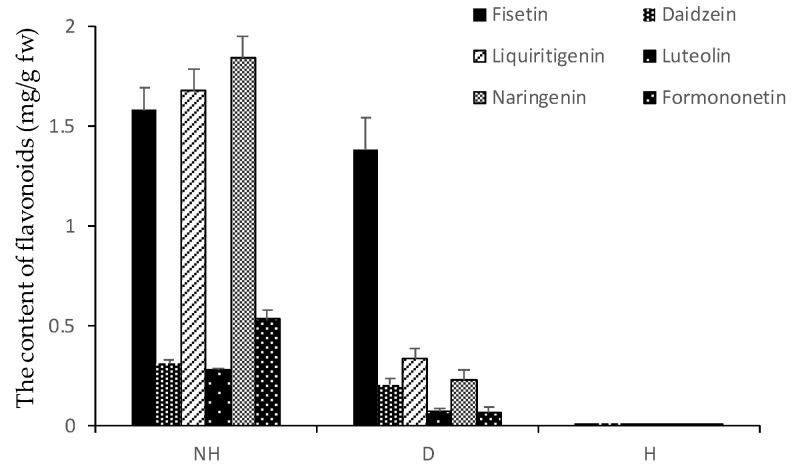
The contents of six flavonoids in natural HW and the D and H zones of pruned stem.

Flavonoids extracted from HW and the D and H zones were also analyzed via HPLC to determine the content of individual flavonoids in these areas (Figure 3). The chromatogram of the HW sample contained 18 major peaks, and all these peaks and two other peaks (16 and 19) were present in the D-zone chromatogram. However, the amounts of all flavonoids, except for one corresponding to peak 2, in the D zone were considerably lower than those in the HW. There was only one peak in the H zone. By comparing each peak’s retention time with that of six commercially available flavonoids in the same chromatographic system (identical mobile and stationary phases), we found peaks 3, 5, 7, 8, 13, and 20 corresponding to fisetin, daidzein, liquiritigenin, luteolin, naringenin, and formononetin, respectively (Figure 3). All six flavonoids were present in the HW and D samples, but their contents in the D zone are significantly lower than those in HW (Figure 4). 

### 3.3. Characterization of Gene Expression Patterns in D and H Through RNA Sequencing

Global gene expression profiles in the D and H zones were analyzed and compared. After stringent data filtering and quality checks, approximately 58 million high-quality clean reads were obtained from the D and H samples with 97.52% and 97.50% Q20 bases, respectively (base quality being more than 20). Transcriptome de novo assembly was carried out using Trinity, a program for assembling short reads. We identified 67,473 unigenes, with 18,297 unigenes having a length of more than 500 bp (Table S1). We queried the NCBI non-redundant protein database (nr) and the Swiss-Prot protein database with all the unigenes through the use of BlastX with the cut-off E-value of 10^−5^. In total, 34,427 and 14,562 unigenes (51.02% and 21.58% of all unigenes) returned matches that exhibit similarities higher than the cut-off E-value in the nr and Swiss-Prot databases, respectively. 

Gene ontology (GO) enrichment analysis was performed to classify the function of the unigenes. The three largest functional categories were biological process (80,672), cellular component (49,042), and molecular function (37,874). Within the biological process category, cellular component organization or biogenesis, metabolic process, and single-organism process were the most common. Within the cellular component category, the majority of the GO terms were assigned to cell and cell junction. For molecular function, most were assigned to binding and catalytic activity (Figure S1). We also assigned the annotated sequences to clusters of orthologous groups (COG), and 16,157 unigenes have a COG classification. Among the 25 COG categories, the cluster for ‘Posttranslational modification, protein turnover, chaperones’ represents the largest group (2013, 12.46%), followed by ‘General function prediction only’ (1906, 11.80%) and ‘Translation, ribosomal structure and biogenesis’ (1674, 10.36%) (Figure S2). We searched the KEGG (Kyoto Encyclopedia of Genes and Genomes) database to analyze which pathways the unigenes belong to. In total, 17,035 sequences were assigned to 19 KEGG pathways. The pathways represented most were Translation (1771, 12.59%) and Carbohydrate metabolism (1723, 12.25%) (Figure S3).

The level of unigene expression was calculated using reads per kb per million reads (RPKM) method, which helped identify up-regulated and down-regulated genes in the H and D zones. Differentially expressed genes (DEGs) with significant changes were determined using Poisson distribution equation, with the threshold of False Discovery Rate (FDR) lower or equal to 0.001 and the absolute value of log_2_ ratio lower or equal to 1. In total, 3621 and 1933 unigenes showed significant differential expression in the H and D zones, respectively (Figure S4).

### 3.4. Wounding Induced the Expression of The Genes Involved in The Phenylpropanoid and Flavonoid Biosynthesis

**Figure 5:**
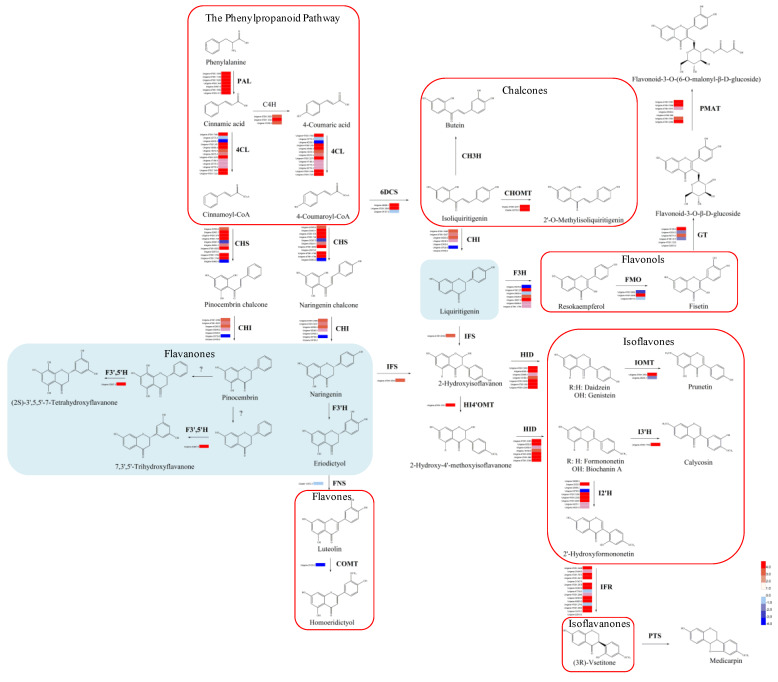
Putative biosynthesis pathways for phenylpropanoid and flavonoid biosynthesis pathways and expression patterns of the genes for these pathways in *D. odorifera.*

To elucidate how wounding impacts the production of phenylpropanoids and flavonoids, we identified and mapped the unigenes of *D. odorifera* that are predicted to be involved in the phenylpropanoid and flavonoid biosynthesis (Figure 5). Twenty-three unigenes appear to be involved in the initial three steps of the phenylpropanoid pathway and encode seven PALs (phenylalanine ammonia lyases), three C4Hs (trans-cinnamate4-monooxygenase), and 13 4CLs (4-coumarate-CoA ligases). The enzymes encoded by these genes catalyze a series of reactions to form cinnamoyl-CoA or 4-coumaroyl CoA, two substrates for the biosynthesis of flavonoids. We also identified 85 candidate unigenes that belong to 18 gene families associated with the flavonoid biosynthesis (Figure 5). These genes are predicted to participate in three main subpathways that utilize cinnamoyl-CoA and 4-coumaroyl CoA. In the upstream of their biosynthetic pathways, we found 11 CHSs (chalcone synthases), seven CHIs (chalcone isomerases), and three 6DCSs (NAD(P)H-dependent 6’-deoxychalcone synthases). These enzymes are involved in a two-step condensation that produces the basic skeletons of some flavonoids, including pinocembrin, naringenin, and liquiritigenin. Following the core subpathway of pinocembrin, we identified one F3’5’H (flavonoid-3’,5’-hydroxylase), an enzyme that produces two flavanones, (2S)-3’,5,5’-7-tetrahydroxyflavanone and 7,3’,5’-trihydroxyflavanone. In the core subpathway of naringenin, one FNS (flavone synthase) and one COMT (flavone 3’-O-methyltransferase) were identified. These enzymes synthesize luteolin and homoeridictyol, respectively. In the core subpathway of liquiritigenin, one IFS (2-hydroxyisoflavanone synthase) and one HI4’OMT (2,7,4’-trihydroxyisoflavanone 4’-O-methyltransferase) were identified, which produce isoflavanones 2-hydroxyisoflavanone and 2-hydroxy-4’-methoxyisoflavanone, respectively. These biosynthesis pathways were catalyzed by seven HIDs (2-hydroxyisoflavanone dehydratases), two IOMTs (isoflavone 7-O-methyltransferases), one I3’H (isoflavone 3’-hydroxylase), nine I2’Hs (isoflavone 2’-hydroxylases), and 15 IFRs (isoflavone reductases), resulting in seven isoflavones, including genistein, daidzein, prunetin, formononetin, biochanin A, calycosin, and 2’-hydroxyformononetin, and isoflavanone (3R)-vsetitone. The synthesis of liquiritigenin is also catalyzed by seven F3Hs (flavonone 3-hydroxylases) and three FMOs (flavonoid 3’-monooxygenases), yielding two flavonols, resokaempferol and fisetin, respectively. Fisetin was catalyzed by six GTs (UDP-glucose flavonoid 3-O-glucosyltransferases) and seven PMATs (phenolic glucoside malonyltransferases), yielding flavonoid-3-O-β-D-glucoside and flavonoid-3-O-(6-O-malonyl-β-D-glucoside), respectively. There were 90 unigenes that showed upregulation, while only 18 unigenes were downregulated in the phenylpropanoid and flavonoid pathways after three weeks of pruning (Figure 5). 

In the phenylpropanoid pathway, pruning increased the expression of most of the unigenes (21 of 23) belonging to three gene families, including PAL, C4H, and 4CL. Similarly, 69 out of 85 unigenes mapped to the flavonoids pathway, including most of the gene families (16 of 18), CHS, CHI, 6DCS, F3’5’H, F3H, FMO, GT, PMAT, CHOMT, IFS, HI4’OMT, HID, IOMT, I2’H, IFR, and I3’H, were also upregulated by pruning.

### 3.5. Changes in Signal Molecules Associated with Plant Defense in Response To Pruning

**Figure 6:**
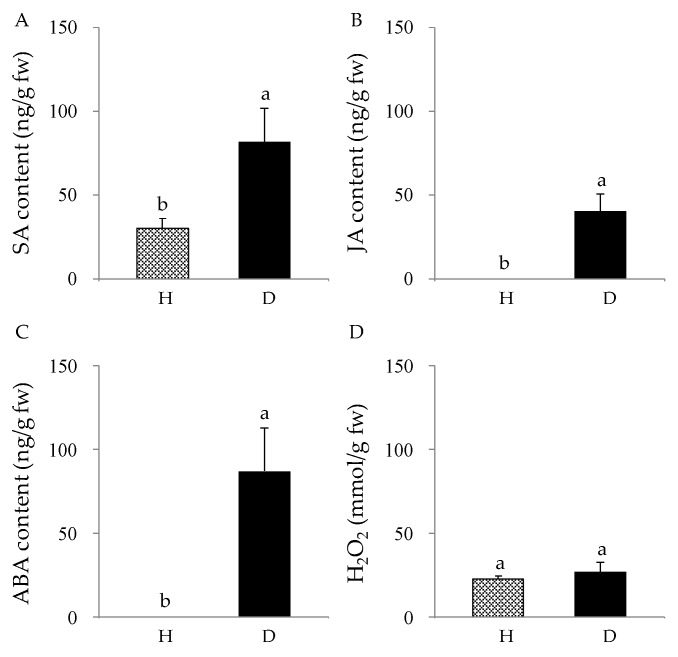
Accumulation of signal molecules in response to pruning. The amounts of SA (**A**), JA (**B**), ABA (**C**), and H_2_O_2_ (**D**) in the D and H zones were compared.

Pruning induced the expression of many genes involved in the phenylpropanoid and flavonoids biosynthesis and the increase of flavonoids content. Since these changes typically occur after wounding, we determined whether pruning increased the accumulation of wounding-associated signal molecules. The contents of ET, JA, H_2_O_2_, SA, and ABA in the D and H zones were quantified (Figure 6). Pruning increased the concentration of JA and ABA in the D zone, while they were not detectable in the H zone. We also detected a 2.7-fold increase in the SA content in the D zone compared to that in the H zone. However, pruning had no significant effect on the accumulation of H_2_O_2_, and no ET was detected in the H and D zones.

### 3.6. Expression Patterns of The Genes Involved in The Production of ET, JA, ABA, ROS, and SA in The D Zone of D. odorifera

**Figure 7:**
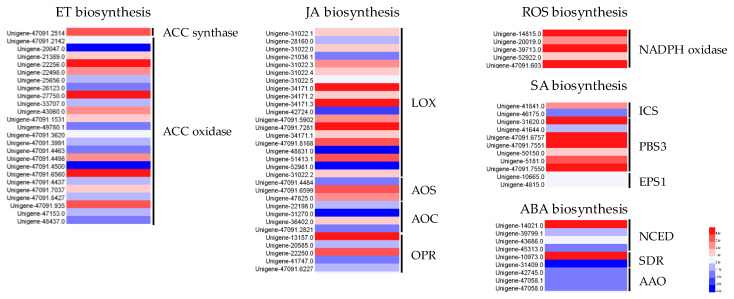
Expression patterns of the genes involved in the production of ET, JA, ABA, ROS, and SA in the D zone.

Based on the results showing that pruning increased the production of JA, ABA, and SA, we analyze the expression patterns of the genes involved in their biosynthesis in the H and D zones. There are 81 unigenes mapped to 13 gene families (ACC synthase, ACC oxidase, LOX, AOS, AOC, OPR, NADPH oxidase, ICS, PBS, EPS, NCED, SDR, AAO), which are involved in synthesizing ET, JA, ABA, ROS, and SA (Figure 7). Forty-seven of 81 unigenes belonging to 11 gene families, except for AAO and EPS gene families, were upregulated. In the ET biosynthesis pathway, 13 unigenes that belong to the ACC synthase (1-aminocyclopropane 1-carboxylate synthase) and ACC oxidase (1-aminocyclopropane-1-carboxylate oxidase) gene families were upregulated. In the JA biosynthesis pathway, 19 unigenes corresponding to LOX (linoleate 13S-lipoxygenase), AOS (allene oxide synthase), AOC (allene oxide cyclase), and OPR (12-oxophytodienoate reductase) were upregulated. In ROS biosynthesis, five unigenes in the NADPH oxidase gene family were upregulated. In the SA biosynthesis pathway, seven unigenes in the ICS (isochorismate synthase) and PBS3 gene families were upregulated. In the ABA pathway, three unigenes in the NCED (9-cis-epoxycarotenoid dioxygenase) and SDR (short-chain dehydrogenase reductase) gene families were upregulated.

### 3.7. Wounding-Associated Signals Induced Flavonoid Biosynthesis in Cell Suspensions of D. odorifera

**Figure 8:**
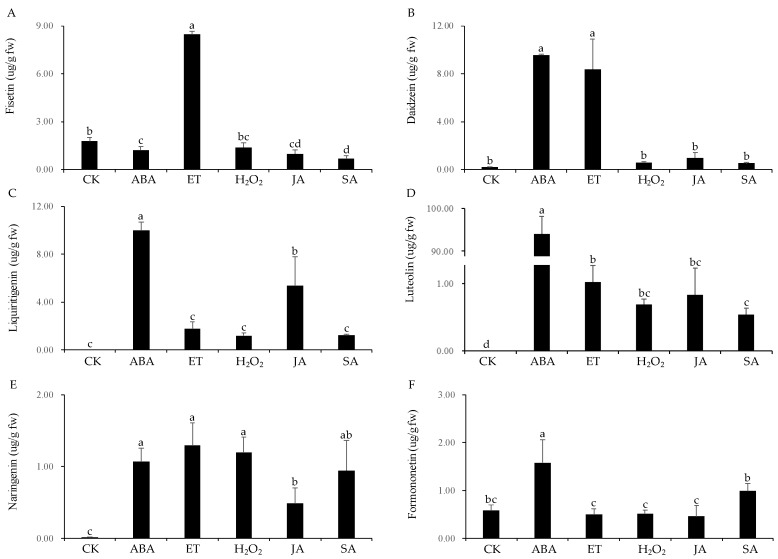
Induction patterns of six flavonoids in response to the treatment of cell suspension cultures with ABA, ET, H_2_O_2_, JA, and SA.

To determine which signals play significant roles in inducing flavonoid biosynthesis in response to wounding, cell suspension cultures of *D. odorifera* were treated with ABA, ET, H_2_O_2_, JA, and SA. All of the molecules increased the production of flavonoids, but their effect on different flavonoids varied. ET induced the production of flavonol fisetin strongest (8.48 μg/g), which is 4.8-fold higher than that in the control. However, the other four signaling molecules had no significant effect on its production (Figure 8A). ABA and ET induced the production of daidzein (isoflavone-type) by 47.3-fold (9.60 μg/g) and 41.4-fold (8.40 μg/g), respectively, while H_2_O_2_, JA, and SA had no significant effect on its production (Figure 8B). ABA and JA induced the production of liquiritigenin (9.99 μg/g and 5.34 μg/g, respectively), while no liquiritigenin was detected in the control. ET, H_2_O_2_, and SA had no significant effect on the production of liquiritigenin (Figure 8C). ABA drastically induced the amount of luteolin (flavone-type), 94.07 μg/g, while no luteolin was detected in the control. The other four molecules also induced the production of luteolin but much lesser degrees (Figure 8D). All molecules were able to significantly induce the synthesis of naringenin (flavanone-type) (Figure 8E). ABA caused 2.7-fold (1.58 μg/g) increase in the production of formononetin (isoflavone-type) compared with that of the control. However, the other four molecules had no significant effect (Figure 8F).

## 4. Discussion

The heartwood of *D. odorifera* has been used for many high-value products. Unfortunately, its overexploitation endangered wild *D. odorifera* trees. Heartwood is a naturally developing part of the xylem in trees, and functions to protect from deterioration caused by insects, marine borers, and microorganisms. The process involved in heartwood formation remains poorly understood in many commercially important species [36]. To induce heartwood formation, abiotic and biotic stresses, such as mechanical wounding [22], drought [37], phytohormones [38], and fungal infection [39], have been used. In this study, we showed that pruning caused the death of ray and axial parenchyma cells and filled the D zone with brownish or dark-brown resin, which marked the transformation of sapwood into heartwood. In the D zone, the vessels filled with brownish or dark-brown resin led to vessel occlusions, which is a common feature of heartwood. Moreover, the discolored wood induced by pruning, which is usually considered wound or pathological heartwood [40], has a similar color and structure to that of natural heartwood (Figure 1). 

Furthermore, the discolored wood formed by pruning contains nearly half of the total flavonoids present in natural heartwood, while there were almost no flavonoids detected in in the N and H zones and in the SW (Figure 2). This result indicates that healthy *D. odorifera* trees do not produce flavonoids, and wounding the tree initiates the production of flavonoids. A comparison of HPLC chromatograms of flavonoids in the discolored wood, heartwood and healthy wood showed that the discolored wood has the highest chemical similarity to natural heartwood (Figure 3). Six major flavonoids (fisetin, daidzein, liquiritigenin, luteolin, naringenin, and formononetin) were detected in both the HW and D zone. However, the discolored wood and HW were very different from the healthy wood, in which no flavonoids were detected. These results suggest that wounding is an efficient, novel technique for inducing flavonoid production in *D. odorifera*. Other studies also showed that wounding is an effective stimulus for activating the phenylpropanoid metabolism, thus promoting a higher accumulation of secondary metabolites [16,17,18,19,20,21,22], Other stimuli, such as fungal infection and drought, could also result in the discoloration of wood in *D. odorifera* [22,37,38,39], but little information is available about how wounding causes the accumulation of flavonoids in the wood.

**Figure 9:**
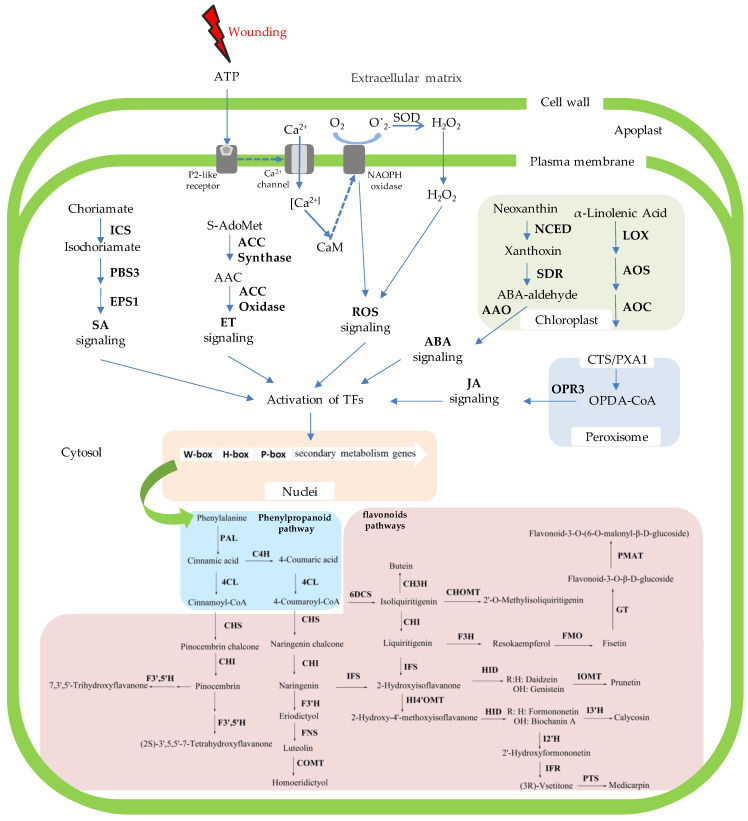
A model summarizing how wound-induced signaling regulates the phenylpropanoid and flavonoid biosynthesis pathways involved in heartwood production in *D. odorifera*.

To remedy this knowledge deficiency, we analyzed the transcriptome data derived from the D and H zones of wounded *D. odorifera* (Figure 5 and Figure 7), which helped decipher the mechanism of wounding-induced flavonoid biosynthesis and their regulation (Figure 9). This is the first report of how *D. odorifera* regulates transcription in response to pruning. The transcriptome data also identified 108 *D. odorifera* genes associated with the phenylpropanoid and flavonoid pathways (Figure 5). Analysis of DEGs show that wounding unregulated the expression of 90 unigenes, which covered 19 gene families (PAL, C4H, 4CL, CHS, CHI, 6DCS, F3’5’H, F3H, FMO, GT, PMAT, CHOMT, IFS, HI4’OMT, HID, IOMT, I2’H, IFR, and I3’H). The transcriptome data generated in this study not only help study how other stimuli affect flavonoid pathways in *D. odorifera,* but also serve as a valuable resource for future research on the biology of *D. odorifera* and the evolution of its metabolism. Resulting insights will help produce valuable bioactive components.

Production of useful plant compounds via the use of cultured cells has some advantages, one of which is their production under sterile and controlled conditions. The system also allows controlled ilicitation of *in vitro* cultures with specific signals to enhance the production of desirable products [41,42,43]. In our study, all signal molecules (ET, JA, H_2_O_2_, SA, and ABA) increased the accumulation of different flavonoids in cell suspensions of *D. odorifera*. ABA significantly induced the production of five kinds of flavonoids (daidzein, liquiritigenin, luteolin, naringenin, and formononetin), reaching a summed concentration of 116.30 μg/g fw. ET also significantly induced four flavonoids (fisetin, daidzein, luteolin, naringenin), resulting in a total concentration of 19.90 μg/g fw. H_2_O_2_, JA, and SA induced the production of luteolin and naringenin, with their total concentrations being 1.88, 1.31, and 1.48 μg/g fw, respectively.

Accumulation of specific secondary metabolites is a common plant response to biotic or abiotic stresses, and plants employ many signaling molecules to regulate and coordinate the accumulation of plant secondary metabolites [44]. Wounding caused by pests and pathogens is a common stress to plants. Plants have evolved constitutive and inducible defense mechanisms to respond to wounding in order to prevent further damage [45]. These plant defense responses involve stress-induced signaling molecules as key regulators [16] Typically, mechanical damage to plants immediately causes a rapid release and activation of apoplastic peroxidases and burst of reactive oxygen species (ROS) [46,47]. Wounding also induces the de novo synthesis of JA [48], ABA [49], ET [50,51], and SA [52], which are known to activate a network of interconnected pathways that coordinate host defense responses [45]. Our data showed that wounding induced the de novo synthesis of JA, ABA, and SA, while no significant changes in the production of H_2_O_2_ and ET in the D zone. However, DEG analysis of the signaling molecule biosynthesis pathways between the H and D zones showed that 58.0% (47 of 81) of unigenes mapped to biosynthesis pathways for all five signaling molecules (ET, JA, ABA, ROS, and SA) were upregulated in the D zone compared to in the H zone. Specifically, 19 unigenes belonging to the LOX, AOS, AOC, and OPR gene families (JA biosynthesis ), three unigenes in NCED and SDR gene families (ABA biosynthesis), and seven unigenes in the ICS and PBS3 gene families (SA biosynthesis) were upregulated, which is consistent with the increased production of JA, SA and ABA upon wounding. However, although 13 unigenes in the ACC synthase and ACC oxidase gene families (ET biosynthesis) and five unigenes in the NADPH oxidase gene family (ROS biosynthesis) were upregulated, we could not detect significant changes in the H_2_O_2_ and ethylene contents (Figure 6). It is known that wounding causes significant increases in H_2_O_2_ and ethylene after several hours, which is why they have been considered as early-signaling molecules. It is quite possible that we failed to detect them because we collected samples three weeks after pruning. Further studies are needed to test whether H_2_O_2_ and ethylene are involved in inducing flavonoid biosynthesis.

Plant secondary metabolites are unique sources for pharmaceuticals, food additives, flavors, and other industrial products [53]. The commercial importance of these secondary metabolites has resulted in great interest in their production and in exploring possibilities of enhancing their production by many means in recent years [54]. Accumulating evidence demonstrates the key roles played by plant signal molecules in regulating secondary metabolites [41]. It is well known that JA is an important signal in regulating biotic and abiotic stresses responses, and JA has been shown to induce the production of several compounds (alkaloids, terpenoid and phenolic phytoalexins, coumarins, and taxanes) in many plant species [55,56]. Salicylic acid, a well-known signal for systemic acquired resistance, is induced in response to infection by many pathogens and can also induce the production of secondary metabolites in plants [42,57,58]. SA can induce the production of more naphtodianthrones and phenylpropanoids in *Hypericum perforatum* suspension cell cultures [42]. ABA can induce the production of indole alkaloids, mono- and sesquiterpenes, anthocyanins, and taxol [43,59,60]. Exposure to ozone caused a rapid increase in the levels of ABA and sequentially enhanced Taxol production in suspension cell cultures of *Taxus chinensis* [59]. ROS, predominantly the superoxide anion (O^2−^) and hydrogen peroxide (H_2_O_2_), mediate the accumulation of secondary metabolites involved in plant-pathogen interactions, such as isoflavones, isoprenoids, phenols or alkaloids, and phytosterols [61]. Ethylene can increase flavonoid, anthocyanin, and stilbenoid production [62]. 

More studies are needed to understand how wounding leads to cellular and molecular changes caused by wounding, but we hypothesize that wounding induces the expression of some genes involved in synthesizing JA (LOX, AOS, AOC, and OPR), ABA (NCED and SDR), SA (ICS and PBS3), ET (ACC synthase and ACC oxidase), and ROS (NADPH oxidase) presumably through secondary messengers, resulting in increased levels of JA, ABA, SA, ET, and H_2_O_2_. These signals regulate the expression or activity of transcription factors (TFs) in nuclei (Figure 9), which leads to the activation of the genes involved in the phenylpropanoid pathway. Most genes of the flavonoid pathway (CHS, CHI, 6DCS, F3’5’H, F3H, FMO, GT, PMAT, CHOMT, IFS, HI4’OMT, HID, IOMT, I2’H, IFR, and I3’H) were upregulated. Previous studies have shown that extracellular ATP accumulated at the site of injury plays a key role in triggering ROS production [63]. Upon the application of wounding stress, cell disruption occurs, inducing the liberation of cytosolic ATP into the extracellular matrix. The released ATP diffuses from the site of injury into adjacent cells, where it is recognized by its receptor at the plasma membrane. Once ATP binds to its receptor, the cytosolic Ca^2+^ concentration is increased, triggering the activation of NADPH oxidase and thus O_2_^•−^ production. Super oxide radical is transformed into H_2_O_2_ by SOD (Figure 9). These ROS (O_2_^•−^ and H_2_O_2_) act as a signal that increase the mitochondria respiration rate in the tissue, inducing higher ROS levels. Simultaneously, ROS activate the phenylpropanoid metabolism, producing phenolic compounds, whereas ET and JA are essential to modulate ROS levels in carrot [18,19]. In broccoli, genes involved in JA biosynthesis, phenylpropanoid pathway, amino acid, and glucosinolate biosynthesis were among the highest upregulated genes in response to wounding [17].

## Supplementary Materials

The following are available online at https://www.mdpi.com/2073-4425/11/5/478/s1, Table S1. Statistics of the results from analyzing the unigenes using multiple public databases, Figure S1. Histogram showing the distribution of the unigenes in Gene Ontology (GO) functional categories, Figure S2. Classification of the unigenes into Orthologous groups, Figure S3. Functional classification and pathway assignment of the unigenes via the use of KEGG, Figure S4. Expression levels of differentially expressed genes in D and H samples.

## Abbreviations

IUCN	The International Union for Conservation of Nature
CITES	Trade in Endangered Species
RNA-Seq	RNA sequencing
N	Necrosis
D	Discolored
H	Healthy
HW	Heartwood
SW	Sapwood
CTF	The content of total flavonoids
C4H	Trans-cinnamate4-monooxygenase
4CL	4-coumarate-CoA ligase
CHS	Chalcone synthase
CHI	Chalcone isomerase
6DCS	NAD(P)H-dependent 6’-deoxychalcone synthase
F3’5’H	Flavonoid-3’,5’-hydroxylase
FNS	Flavone synthase
COMT	Flavone 3’-O-methyltransferase
IFS	2-hydroxyisoflavanone synthase
HI4’OMT	2,7,4’-trihydroxyisoflavanone 4’-O-methyltransferase
HID	2-hydroxyisoflavanone dehydratases
IOMT	Isoflavone 7-O-methyltransferase
I2’H	Isoflavone 2’-hydroxylase
IFR	Isoflavone reductase
F3H	Flavonone 3-hydroxylase
FMO	Flavonoid 3’-monooxygenase
I3’H	Isoflavone 3’-hydroxylase
GT	UDP-glucose flavonoid 3-O-glucosyltransferase
PMAT	Phenolic glucoside malonyltransferase
ACC synthase	1-aminocyclopropane 1-carboxylate synthase
ACC oxidase	1-aminocyclopropane-1-carboxylate oxidase
LOX	Linoleate 13S-lipoxygenase
AOS	Allene oxide synthase
AOC	Allene oxide cyclase
OPR	12-oxophytodienoate reductase
ICS	Isochorismate synthase
NCED	9-cis-epoxycarotenoid dioxygenase
SDR	Short-chain dehydrogenase reductase
AAO	Abscisic-aldehyde oxidase
ROS	Reactive oxygen species
NR	Nonredundant
KEGG	The Kyoto Encyclopedia of Genes and Genomes
COG	The Clusters of Orthologus Groups
GO	The Gene Ontology
