# Supplementary material for: Molecular Mechanism Underlying Mechanical Wounding-Induced Flavonoid Accumulation in Dalbergia odorifera T. Chen, an Endangered Tree That Produces Chinese Rosewood
Source: Genes (Basel). 2020 Apr 28;11(5):478. doi: 10.3390/genes11050478 (PMC7291145; doi:10.3390/genes11050478)
Supplement: Supplementary file 1 [file genes-11-00478-s001.pdf]

Supplementary Data

Table S1. Statistics of the results from analyzing the unigenes using multiple public databases

Public databases	Number of unigenes	Percentage of unigenes
NR	34427	51.02%
NT	32373	47.97%
SwissProt	14562	21.58%
ALL	67473	100.00%

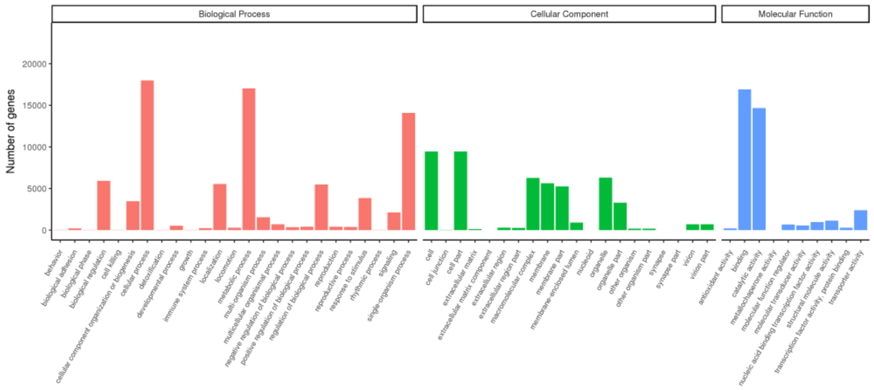

Figure S1. Histogram showing the distribution of the unigenes in Gene Ontology (GO) functional categories.

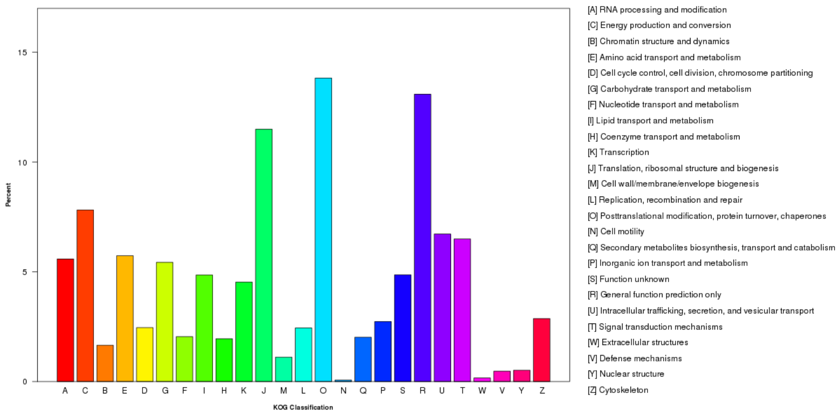

Figure S2. Classification of the unigenes into Orthologous groups.

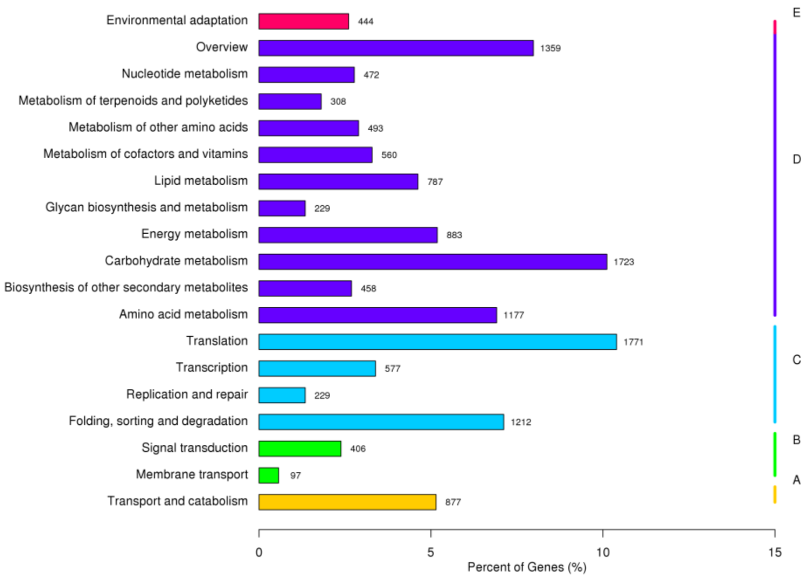

Figure S3. Functional classification and pathway assignment of the unigenes via the use of KEGG.

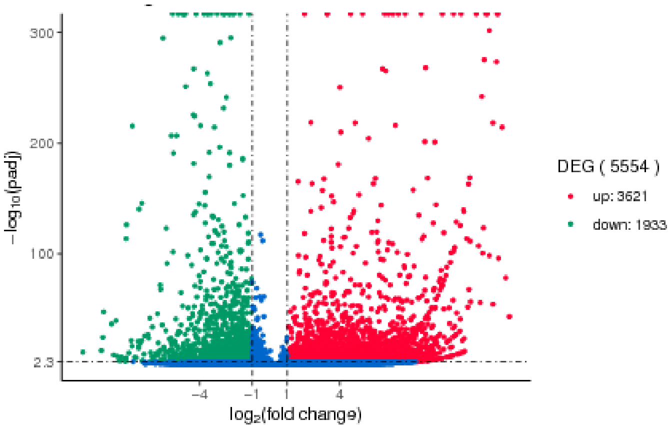

Figure S4. Expression levels of differentially expressed genes in D and H samples.

Up-regulated and down-regulated genes are denoted by red and green dots, respectively. Blue dots correspond to those that are not differentially expressed.
